# GIS based analysis of Intercity Fatal Road Traffic Accidents in Iran

**Published:** 2015

**Authors:** A Alizadeh, M Zare, M Darparesh, S Mohseni, M Soleimani-Ahmadi

**Affiliations:** * Department of Public Health Social Determinants in Health Promotion Research Center, Hormozgan University of Medical Sciences, Bandar Abbas, Iran; ** Department of Occupational Health Engineering, Faculty of Health, Hormozgan University of Medical Sciences, Bandar Abbas, Iran; *** Research Center for Social Determinants of Health Promotion, Hormozgan University of Medical Sciences, Bandar Abbas, Iran; **** Department of Public Health, Faculty of Health, Hormozgan University of Medical Sciences, Bandar Abbas, Iran; ***** Department of Medical Entomology and Vector Control, Faculty of Health, Hormozgan University of Medical Sciences, Bandar Abbas, Iran

**Keywords:** GIS, Intercity Fatal Accidents, Iran

## Abstract

Road traffic accidents including intercity car traffic accidents (ICTAs) are among the most important causes of morbidity and mortality due to the growing number of vehicles, risky behaviors, and changes in lifestyle of the general population. A sound knowledge of the geographical distribution of car traffic accidents can be considered as an approach towards the accident causation and it can be used as an administrative tool in allocating the sources for traffic accidents prevention. This study was conducted to investigate the geographical distribution and the time trend of fatal intercity car traffic accidents in Iran.

To conduct this descriptive study, all Iranian intercity road traffic mortality data were obtained from the Police reports in the Statistical Yearbook of the Governor’s Budget and Planning. The obtained data were for 17 complete Iranian calendar years from March 1997 to March 2012. The incidence rate (IR) of fatal ICTAs for each year was calculated as the total number of fatal ICTAs in every 100000 population in specified time intervals. Figures and maps indicating the trends and geographical distribution of fatal ICTAs were prepared while using Microsoft Excel and ArcGis9.2 software.

The number of fatal car accidents showed a general increasing trend from 3000 in 1996 to 13500 in 2012. The incidence of fatal intercity car accidents has changed from six in 100000 population in 1996 to 18 in 100000 population in 2012. GIS based data showed that the incidence rate of ICTAs in different provinces of Iran was very divergent. The highest incidence of fatal ICTAs was in Semnan province (IR= 35.2), followed by North Khorasan (IR=22.7), and South Khorasan (IR=22). The least incidence of fatal ICTAs was in Tehran province (IR=2.4) followed by Khozestan (IR=6.5), and Eastern Azarbayejan (IR=6.6). The compensation cost of fatal ICTAs also showed an increasing trend during the studied period.

Since an increasing amount of money was being paid yearly for the car accidents, which were in their nature preventable, the key players in road safety including governments, car manufacturers, and road developers were recommended to use GIS based accident data for a more efficient planning and budgeting towards the intercity car accidents reduction.

## Introduction

Road traffic accidents including city car traffic accidents and intercity car traffic accidents (ICTAs) are among the most important causes of morbidity and mortality, due to the growing number of vehicles, risky behaviors, and changes in lifestyle of the general population [**2**]. According to the global status report on road safety in 2013, by WHO, the total number of road traffic deaths around the world remained unacceptably high at 1.24 million per year [**1**]. Although some researchers have reported an enormous growth in the number of motor vehicles as a main cause of accidents [**2**], surprisingly 97% of the road accidents occur in developing countries, which have only 48% of vehicles [**2**]. This fact emphasized on the multifactor nature of the accident causes, which should be considered by researchers and experts in the accident analysis and causation.

While the WHO reported five factors including drinking and driving, speeding, and failing to use motorcycle helmets, seat-belts, and child restraints as the key risk factors of road traffic accidents, there are many elements that should be considered in road traffic accident analysis. For example, a study reported that the separation and divorce are associated with a 2.9 fold increase in serious injury road traffic risk [**20**]. Another study revealed that speed cameras have a significant effect on reducing accidents up to 200 meters from the camera sites [**4**,**5**]. A study in Greece showed that police enforcements could play an important role in the rate of road accidents [**7**]. The consumption of drugs and alcohol is also associated with serious car accidents [**16**]. In addition, according to the report of the International Commission on Illumination, installation of road lighting is expected to reduce nighttime injury accidents to 30 percent [**8**]. An important factor, which should be considered in road traffic accident fatality analysis, is poor infrastructure in developing countries in the form of bad roads and inadequate access to healthcare.

Currently, among the 100 important causes of death, traffic accidents are situated on the ninth position. However, some predictions indicated that traffic accidents as a cause of death will move on the sixth place and in terms of disability-adjusted life years’ (DALYs) and years of life lost (YLL) will be on the third and second place respectively by the year 2020 [**2**,**3**]. The burden of road traffic accidents extends far beyond their financial implications and includes different social and psychological impacts on the society. Many articles in literature have reported different psychosocial consequences of road traffic accidents including post-traumatic stress disorder which has been observed in 10 to 50 percent of people who were involved in a road traffic accident [**6**,**9**]. The study of Hours et al. also showed a wide range of chronic consequences for people who were injured in traffic accidents including impact on everyday life of their family, impact on leisure, projects, emotional life, and job, relational difficulty in the couples, impaired sexual life, increased rate of separation, and post-traumatic stress disorder [**10**,**11**].

The numbers of road accidents, fatalities, and injuries are being reported annually in different parts of the worldly different institutions and governments that are considered important indicators of the road safety situations. But, these statistics are not detailed enough to show the main causes or risk factors generating the road accident problems, and more importantly do not indicate what countermeasures should be applied to reduce the number of road accidents. A proper identification of the causes of the road traffic accidents can be achieved by a detailed analysis of the related statistics.

A sound knowledge of geographical distribution of car traffic accidents can be considered an approach towards accident causation and it can be used as an administrative tool in allocating the sources for traffic accidents prevention. This study was conducted to investigate the geographical distribution and the time trend of fatal intercity car traffic accidents in Iran. The results of this study can be used in the improvement of future planning and efficient budgeting to reduce the rate of car accidents.

## Method

To conduct this descriptive study, all the Iranian intercity road traffic mortality data were obtained from the Police reports in the Statistical Yearbook of the Governor’s Budget and Planning. The obtained data were for 17 complete Iranian calendar years from March 1997 to March 2012. The total number of fatal ICTAs in each year during this time period was determined to clarify the time trend fatal ICTAs. In addition, the incidence rate (IR) of fatal ICTAs for each year was calculated as the total number of fatal ICTAs in every 100000 population at specified time intervals. As the focus of this study is on the geographical distribution of intercity car accident mortality, the incidence of intercity car accident death was calculated for 30 provinces of Iran in the studied period.

Figures and maps indicating the trends and geographical distribution of fatal ICTAs were prepared by using Microsoft Excel and ArcGis9.2 software.

## Results

**Fig. 1:**
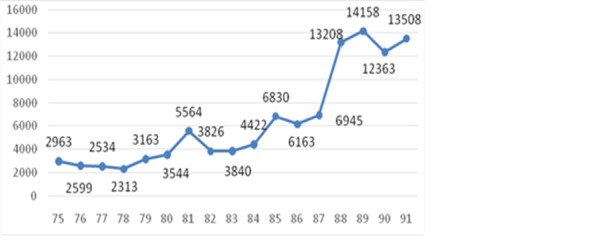
Total number of fatal intercity car accidents happening each year during 1996 and 2012

The total number of fatal ICTAs in each year during the studied period is shown in **Fig. 1**. According to this figure, the number of fatal car accidents showed a general increasing trend from 3000 in 1996 to 13500 in 2012. As **Fig. 1** indicated, there was a critical point in the number of ICTAs in the studied time interval. The number of fatal car accidents had dramatically increased from 7000 in 2008 to 13200 in 2009 and to over 14000 in 2010.

**Fig. 2:**
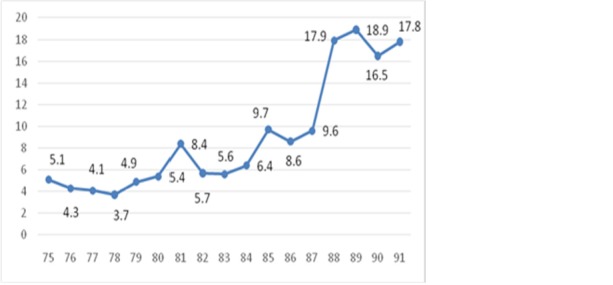
Incidence of intercity fatal car accidents during 1996 and 2012

The incidence of fatal ICTAs during 1996 and 2012 is shown in **Fig. 2**. An increasing trend in the incidence of fatal ICTAs can be seen in this figure so that the incidence of fatal intercity car accidents has changed from six in 100000 population in 1996 to 18 in 100000 population in 2012. The critical point in this figure can also be seen where the incidence of fatal intercity car accidents has increased from 12 in 100000 population to 28 in 100000 population during 2009 and 2011.

**Fig. 3:**
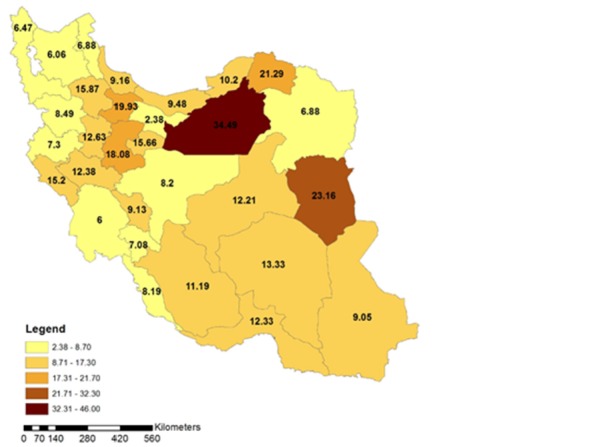
Geographical distribution of the intercity fatal car traffic accidents incidence rate in different provinces of Iran during 1996 and 2012

The geographical distribution of fatal ICTAs during the studied period is shown in **Fig. 3** in terms of the average of fatal ICTAs per 100000 population in different provinces. According to this figure, the highest incidence of fatal ICTAs is in Semnan province (IR= 35.2), followed by North Khorasan (IR=22.7), South Khorasan (IR=22), Qazvin (IR=20.5), and Markazi (IR=19.1). The lowest incidence of fatal ICTAs is in Tehran province (IR=2.4) followed by Khuzestan (IR=6.5), Eastern Azerbaijan (IR=6.6), Western Azerbaijan (IR=7.1), Ardebil (IR=7.2), Kermanshah (IR=7.9), and RazaviKhorasan (IR=8).

**Fig. 4:**
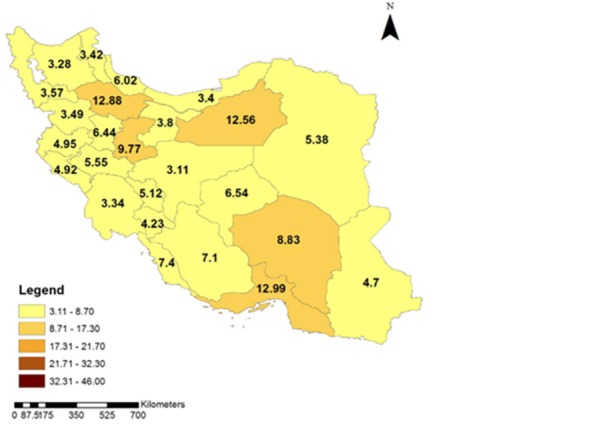
Geographical distribution of the intercity fatal car traffic accidents incidence rate in different Provinces of Iran in 1996

**Fig. 5:**
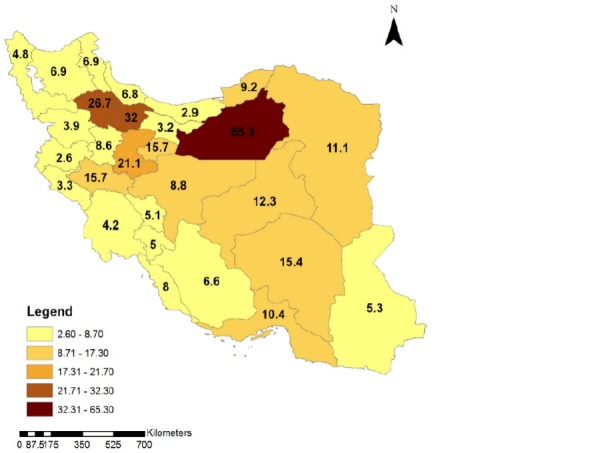
Geographical distribution of the intercity fatal car traffic accidents incidence rate in different provinces of Iran in 2002

**Fig. 6:**
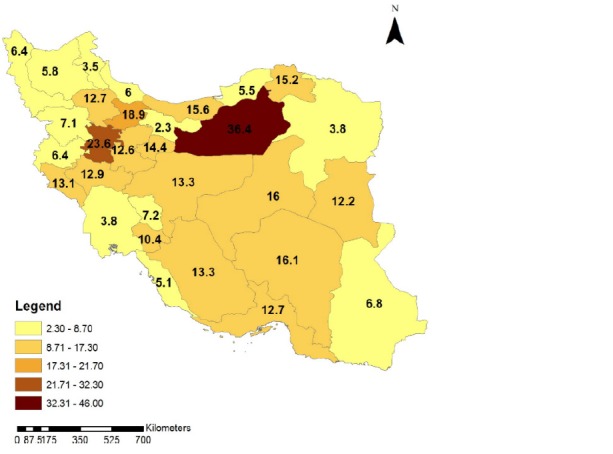
Geographical distribution of the intercity fatal car traffic accidents incidence rate in different provinces of Iran in 2007

**Fig. 7:**
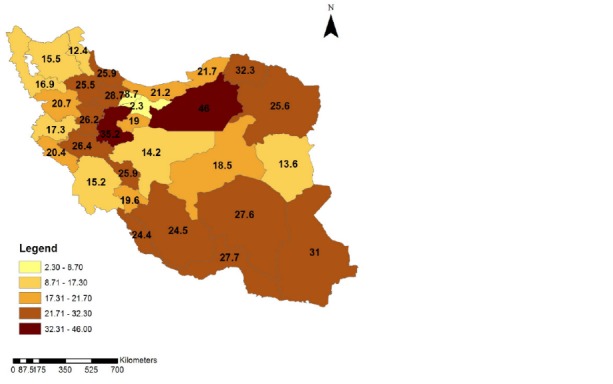
Geographical distribution of the intercity fatal car traffic accidents incidence rate in different provinces of Iran in 2012

To clarify the time trend of the intercity car traffic accidents in different provinces of Iran during 1996 and 2012, the geographic distribution of fatal car accidents incidence rate is illustrated in **Fig. 4**-**7**. These figures illustrate the fatal car accidents incidence rates in 1996, 2002, 2007, and 2012 in different provinces of Iran.

**Fig. 8:**
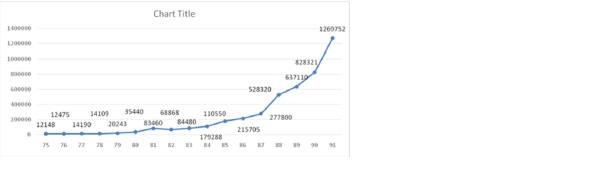
Compensation costs of fatal intercity car traffic accidents during 1996 and 2012

The compensation cost of fatal ICTAs was also considered in this study. **Fig. 8** shows the increasing trend of IFCA compensation costs during 1996 and 2012. A critical event can also be seen in this picture during 2011 and 2012, where the compensation costs have increased from 828321 million Rials to 1269752 million Rials.

## Discussion

This study was conducted to investigate the geographical distribution and the time trend of fatal ICTAs in Iran during 1996 and 2012. Our results showed an increasing trend in the incidence of fatal ICTAs during the studied period. This trend was parallel with the trend of car accidents fatalities in the whole world as studies reported the annual number of road traffic deaths to be 300000 in the late 1970s and 1240000 in 2013 (1 and 5). Such an increasing trend can be due to the increased motorization and mileage, risky behaviors, and changes in lifestyle of general population (2 and 11). Although the increased number of vehicles plays an important role in increasing the trend of fatal road traffic accidents, it seems that some more important factors play their roles in this increasing trend. According to WHO latest investigations, 80% of the world’s road traffic deaths occur in the middle-income countries that only have half of the world’s vehicles [**1**]. These statistics emphasized that the factors other than an increased number of vehicles have dominant roles in increasing the rate of road traffic fatalities in the developing countries. Some of these factors, especially in the developing countries, may originate in poor infrastructures including bad roads, inadequate access to healthcare facilities, low road to vehicle ratio.

The focus of this study was on the geographical distribution of fatal ICTAs in Iran to find the hot spots that have more effects on increasing the number of accidents. According to our results, the average of the fatal ICTAs incidence in all the provinces of Iran during the studied period was 9. In this period, Semnan province had the highest fatal ICTAs incidence rate among 30 provinces of Iran, with an average incidence rate of 35.2 (**Fig. 3**). This incidence rate was nearly four times more than the average of the fatal ICTAs incidence rate in all of the provinces of Iran. Surprisingly, Tehran province, which is the capital of Iran, with the highest expected intercity travels, had the lowest fatal ICTAs incidence rate (IR=2.4), which is nearly four times less than the average of the fatal ICTAs incidence rate in all of the provinces of Iran during the studied period. A simple comparison revealed that the average of the incidence rate of fatal ICTAs in Semnan province was nearly 15 times more than that in the Tehran province. Exploring the reasons for such a wide difference between the incidence rates of fatal car accidents in the two provinces of a unique country will be very beneficial for accident causation analysis and source allocation towards the lowering of the car accident rates. Tehran is the capital of Iran, and there should be more travels from other provinces to Tehran and from Tehran to other provinces and the highest incidence rate of fatal ICTAs was expected to be in Tehran province. The reason for such low incidence of fatal ICTAs in Tehran province may be the standard and well-illuminated one-way freeways that start from and ends in Tehran. On the other hand, Semnan acts as a bridge between Tehran and Esfahan, which, after Tehran, is the most industrialized and economically important city of Iran. Hence, the high rate of intercity travels is expected to occur in Semnan-Tehran and Semnan-Esfahan roads while the roads of Semnan province are not as good as Tehran or Esfahan roads and it may be the reason for the high rate of fatal ICTAs in Semnan province. The results of this part of the study can be used by governments for efficient budget allocation towards the reduction of fatal ICTAs. In this regard, it seems that more money should be allocated to Semnan province and other provinces that are colored in brown in **Fig. 3** for the implementation of analytical investigations on fatal ICTAs and the promotion of road safety standards.

An increasing trend in fatal ICTAs during 1996 and 2012 can be observed in **Fig. 2**. **Fig. 4**-**7** showed that this increasing trend exists in all provinces of Iran, where the indicating color of all provinces has changed from light yellow towards dark green over the time. Since WHO reported that 80% of the fatal ICTAs occur in countries which have only half of the world’s vehicles [**1**], such an increasing trend is not attributable only to the increased motorization and mileage over the time and there should be more important factors for consideration. In this regard, some researchers suggested the human error as the most common cause of all road traffic accidents [**12**]. In addition, the use of drugs and alcohol can be major risk factors in fatal car traffic accidents [**15**,**20**,**21**]. Philips and Sagberg also found an association between sleep behind the wheel, which may lead to car accident and driving further per year and being younger [**13**]. Hence, road safety campaigns which use personal communication and roadside delivery of a message (fixed or variable message signs, posters, and billboards) targeting young inexperienced drivers will be very effective in the reduction of fatal ICTAs [**14**]. In addition, the implementation of the early education of the road safety in the early stages during school age would be a priority for the reduction of car traffic accidents. Also, the other contributing factors in road safety including surface properties of pavements (such as skid resistance and texture depth), climate, annual average daily traffic, number of lanes on the road, percentage of heavy vehicles on the road, smoking, use of seat belts, and gradients of descending roads should be considered [**17**,**18**,**19**,**22**,**23**].

 As it was expected, from the incidence rates of fatal ICTAs during the studied period, an increasing trend was also found in the compensation costs of the fatal ICTAs over the time (**Fig. 8**). According to this figure, the financial burden of fatal ICTAs in terms of compensation costs which are only one of the direct car accident costs, has increased 5.87 times during 17 years (the effect of inflation was removed) and considering the increasing trend (**Fig. 8**) it will be very much more in the coming years. It means that an increasing amount of money is being paid for the car accidents that are in their nature preventable. Moreover, it should be considered that in addition to direct costs of car accidents such as compensation costs, there may be indirect costs such as psychological suffering and decreased quality of life for both victim and family, which in most cases cannot be compensated.

Regarding the results of this study, key players in road safety including governments, car manufacturers, and road developers are recommended to use GIS based accident data that show high-risk roads for a more efficient planning towards intercity car accidents reduction.

## Conclusion

This study showed an increasing trend in the rate and compensation costs of fatal ICTAs. In addition, GIS based data showed that the incidence rate of ICTAs in different provinces of Iran is very divergent so that the incidence rate of fatal ICTAs in Semnan province is nearly 15 times more than that in Tehran province. Since an increasing amount of money is being paid yearly for the car accidents that are in their nature preventable, key players in road safety including governments, car manufacturers, and road developers are recommended to use GIS based accident data for more efficient planning towards intercity car accidents reduction.
